# Targeting the NLRP3 inflammasome in cochlear macrophages protects against hearing loss in chronic suppurative otitis media

**DOI:** 10.1186/s12974-024-03212-6

**Published:** 2024-09-14

**Authors:** Viktoria Schiel, Ritwija Bhattacharya, Ankur Gupta, Kourosh Eftekharian, Anping Xia, Peter L Santa Maria

**Affiliations:** Department of Otolaryngology-Head and Neck Surgery, School of Medicine, Stanford University, Palo Alto, CA 94305 USA

**Keywords:** Chronic suppurative otitis media, Sensorineural hearing loss, Inflammation, macrophages, NLRP3

## Abstract

The activation of the NLRP3 inflammasome has been linked to several inflammatory and autoinflammatory diseases. Despite cases of potential hearing improvement in immune-mediated diseases, direct evidence of the efficacy of targeting this mechanism in the inner ear is still lacking. Previously, we discovered that macrophages are associated with Sensorineural Hearing loss (SNHL) in Chronic Suppurative Otitis Media (CSOM), the leading cause of this permanent hearing loss in the developing world and incurring costs of $4 to $11 billion dollars in the United States. However, the underlying mechanism remained unknown. Here, we investigate how macrophages drive permanent hearing loss in CSOM. We first confirmed the occurrence of NLRP3 inflammasome activation in cochlear macrophages in CSOM. We then revealed that Outer Hair Cells (OHCs) were protected in CSOM by macrophage depletion and subsequently confirmed the same protection in the NLRP3 knockout condition. Furthermore, we showed that therapeutic inhibition of NLRP3 inflammasome activation and downstream inhibition of IL-1β protects OHCs in CSOM. Collectively, our data demonstrates that the main driver for hearing loss in CSOM is NLRP3 inflammasome activation in cochlear macrophages and this is therapeutically targetable, leading the way for the development of interventions to prevent the leading cause of permanent hearing loss and a costly disease in the developed world.

**Supplementary Information:**

The online version contains supplementary material available at 10.1186/s12974-024-03212-6.

## Introduction

CSOM is a chronic infectious disease affecting approximately 330 million people worldwide and is the most common cause of permanent hearing loss among children in the developing world [1]. It is responsible for an annual economic healthcare burden of 4–11 billion dollars in the United States, remaining a significant unmet medical need [2]. CSOM is caused by bacterial persister cells in biofilms [3] and is recalcitrant to medical and surgical therapy [2, 4]. Because of lack of curative treatments, patients suffer a gradual loss of sensorineural hearing [2]. Currently no treatments exist to protect the hearing in the CSOM ear. Until now, the current thinking that bacterial invasion or bacterial “toxin” induced injury was the cause of SNHL in CSOM.

Previously, we developed a novel CSOM mouse model that has been validated as representing the human disease [5]. In this model we discovered that SNHL in the high frequency region occurs between day 7 and 14 and that SNHL is associated with macrophages in the cochlea [6]. We ruled out direct bacterial invasion and direct hair cell toxicity from ototoxic exposure from the middle ear, leaving a macrophage associated mechanism at the primary cause [6]. It was unknown if the macrophages are the cause of SNHL or reacting to the injury in CSOM.

Macrophages serve as part of the front line of innate immune defense in the cochlea. There is a resident macrophage population residing in the basilar membrane, osseous spiral lamina, spiral ganglion, spiral ligament, and stria vascularis [7–11]. Many studies have shown that macrophages migrate into the cochlea in response to damage caused by noise exposure, ototoxic drug treatment and age-related degeneration [9, 12–16]. However, the exact role of these macrophages after cochlear injury is still unclear.

There is data emerging that the NLRP3 inflammasome may be linked to hearing loss in a number of conditions including genetic hearing loss and vestibular schwannoma associated hearing loss [17]. However, there is a lack of direct evidence of this target in the inner ear. The NLRP3 inflammasome is an innate immune sensor, expressed in immune cells including monocytes, macrophages and dendritic cells. NLRP3 consists of an amino-terminal pyrin domain (Pycard), a central nucleotide-binding oligomerization domain (NACHT domain), and a C-terminal leucine-rich repeat (LRR) domain at the C terminus [18]. The NLRP3 inflammasome can be activated by a variety of signals including microbial components, toxins, and other danger signals. The activated NLRP3 undergoes oligomerization and recruits the adapter protein Pycard to form a complex. Within the inflammasome, procaspase-1 is then cleaved into its active enzyme form caspase-1. Active Caspase-1 cleaves pro-inflammatory cytokines such as interleukin-1β (IL-1β) and interleukin-18 (IL-18) into their mature forms which are then released into the extracellular space [19–22]. The NLRP3 knock out mouse (NLRP3^−/−^) is generated by removing all NLRP3 exons and replacing it with the neomycin resistance gene [23]. It was shown that the IL-1β protein levels were significantly reduced in NLRP^−/−^ peritoneal macrophages in response to bacterial peptidoglycans. We hypothesize that cochlear macrophages drive hair cell injury through NLRP3 inflammasome activation in CSOM. Furthermore, we hypothesize that this mechanism is targetable to prevent SNHL in CSOM. This changes the thinking away from bacterial invasion, or bacterial “toxin” induced injury towards a new immune mediated hypothesis. It also reveals a new therapeutic direction to address the leading cause of permanent hearing loss in the developing world and one that drives significant health care costs in the developed world.

## Materials and methods

### Animals and ethics approval

All animal procedures were approved by the Institutional Animal Ethics Committee (IACUC) at Stanford University. 6- to 8-week-old wild-type C57BL/6J mice (Ca# 000664 JAX, Bar Harbor, ME USA) and NLRP3 mutant mice (Ca# 021302 JAX Bar Harbor, ME USA) were obtained and crossbred. Littermates were used for all experiments and housed in the Stanford University animal care facility with ad libitum access to food and water. Mice procedures were performed under anesthesia using ketamine (80–100 mg/kg) and xylazine (8–10 mg/kg).

### PLX5622 preparation and treatment

PLX5622, the inhibitor of colony stimulating factor 1 receptor, was purchased from Chemgood (4908 Dominion Blvd, Suite F, Glen Allen, VA 23060, USA) and incorporated into diet chow at a concentration of 1200 mg PLX5622/kg, with a corresponding control diet chow lacking PLX5622. The diet chows were obtained from Research Diets, Inc (20 Jules Lane| New Brunswick, NJ 08901 USA). The mice were then administered either the PLX5622-containing chow or the control chow separately for a duration of 14 days.

### Preparation of PAO1 persisters

Pseudomonas aeruginosa (PAO1) with constitutive expression of a chromosomal-encoded luminescence reporter (PAO1.lux) was constructed as previously described [6]. 20 µl of the glycerol stocks of PAO1 was added to 50 ml of Luria-Bertani broth (LB) media and grown overnight at 37 °C in a shaker. The culture from the LB was streaked on a petri dish containing LB Agar medium and incubated overnight at 37 °C incubator. A single isolated colony was picked from the plate and again grown overnight in 10 ml of LB at 37 °C in a shaker maintaining aerobic conditions. The minimum inhibitory concentration (MIC) of Ofloxacin antibiotic was detected against PA01. Bacteria were grown at 37ºC overnight in a 96-well plate where ofloxacin was serially diluted in LB media. To generate persister cells of PAO1, the bacteria were continued growing in the 10 ml of LB at 37 °C in a shaker for 30 h to reach the stationary phase. After 30 h, Ofloxacin was added to the medium of LB. Bacteria were treated with Ofloxacin at 5 times the MIC for 5 h. The medium was centrifuged and washed three timed with PBS at 10,000 rpm for 5 min. The persister pellets was resuspended in PBS. To determine the persisiter cell concentration, the persisters were serially diluted and placed on Agar medium. The CFU was counted after 48 h culture. A concentration of 3.7 × 10^6 CFU/ml was used for all the experiments.

### Chronic suppurative otitis media model

We adapted our validated model of CSOM [6]. In brief, mice were anesthetized and placed on the surgical stage under the microscope. Subsequently, subtotal tympanic membrane (TM) perforation of the left ear was performed, followed by inoculation of 5 µL of PCs into the middle ear cavity at a concentration of 3.7 × 10^6 CFU/ml. All inoculations were consistently performed between 9 and 10 am in our experiments. The middle ear observation at 7 days revealed middle ear effusion, middle ear inflammation, and TM perforation. ~44.5% of TM perforation and middle ear effusion persisted at 14d. However, 55.5% of TM perforation were found to be closed at 14d. To assess this, we poked the TM and observed the middle ear effusion and inflammation persisted. We defined cases where the TM perforation persisted, and the TM reopened at 14d as CSOM. This same method was employed to inoculate 5µL of 1X PBS into the middle ear cavity, serving as the control group. Mice were maintained in a prone position for 30 min following inoculation. The mice were used for all experiments at different time points.

### CFU count for the middle ear

The concentration of bacteria was measured in the middle ear of the mice after generating CSOM. Mice were euthanized, and the middle ear was dissected at 7d after the persisters inoculation. Each middle ear tissue was weighed and homogenized. The supernatant was collected after centrifuging at 1500 rpm for 5 min. Then the supernatant was serially diluted and placed on Agar medium. The CFU was counted after 48–72 h culture and calculated as CFU/ml per middle ear weight.

### Therapeutic treatment

CSOM in mouse was generated immediately after stopping PLX5622 treatment for 14 days, as described in the method above. Cohorts of mice were administered Intraperitoneally with the NLRP3 inflammasome inhibitor MCC950 (Invivogen, Ca# inh-mcc. San Diego, CA 92121 USA) at a concentration of 20 mg/kg (*n* = 7) or the IL-1 receptor antagonist Anakinra (MedChemExpress, Monmouth Junction, NJ 08852, USA) at a concentration of 30 mg/kg (*n* = 8), respectively. The control CSOM group received PBS injections (*n* = 6). Daily treatments started on day 2 after creating the CSOM model at day 0 and continued for 12 days. Rugular diet was.

provided during these therapeutic treatments. Mice cochleae were dissected on day 14 and prepared for both wholemount and cryosection analyses.

### Histological preparations

Cochleae were dissected at 7 days (7d) and 14 days (14d) after middle ear infection. Dissected specimens were fixed in 4% paraformaldehyde (Electron Microscopy Sciences, Hatfield, PA, USA) at 4^0^C overnight. Samples were then decalcified in 0.5 M EDTA (VWR, Radnor, PA) for 48 h at 4^0^C, and washed three times in PBS (Fisher Scientific). For whole-mount preparations, the organs of Corti were dissected out from the cochleae under a stereo microscope. The cochlear epithelium was divided into three parts: apex (70–100% from the base), middle (30–70% from the base), and base (0–30% from the base). For cryosection preparation, cochleae were immersed in a sucrose gradient (10–30%) and embedded in OCT. Samples were collected in 10 μm sections.

### Immunohistochemistry

Whole mount tissues or cryosections were blocked with 5% donkey serum, 0.1% Triton-X 100, 1% BSA, and 0.02% sodium azide (NaN3) in PBS at pH 7.4 for 1 h at room temperature (RT). Samples were then incubated in primary antibodies overnight at 4 °C. The following primary antibodies were employed: rabbit anti-myosin VIIa (1:200; 25–6790; Proteus BioSciences) for whole mount tissues, mouse anti-F4/80 (1:150, # 14-4801-82 Thermo Fisher Scientific, Waltham, MA, USA) for cryosections. The specimens were incubated with secondary antibodies diluted in 0.1% Triton-X 100, 0.1% BSA and 0.02% NaN3 in PBS for 1 h at RT. The secondary antibodies were conjugated with Alexa Fluor 546, and 594 (1:500; A10040, A21209, Thermo Fisher Scientific, Waltham, MA, USA). After washing with PBS, specimens were mounted in ProLong^®^ Gold Antifade Reagent with DAPI (Cell signaling, #8961 Danvers, MA 01923) and placed under a cover slip. Images were captured using a LSM700 confocal microscope (Zeiss, Germany) at 10X magnification. OHCs labeled with myosin VIIa were counted at 75% from the base (apical turn), 40% from the base (middle turn), and 5% from the base (basal turn) along the cochlea using ImageJ software (https://imagej.net/ij/). Macrophages labeled with F4/80 were counted on cryosections.

### Cytokine and chemokine multiplex assay

The cochleae from both the PLX5622 treatment group and control group were dissected out at 14d following PLX5622 treatment. Samples were homogenized mechanically in lysis buffer. This buffer included 1% Triton-X 100 (9002-93-, Sigma-Aldrich, St. Louis, MO, USA), 0.5% NP-40 (FNN0021, Thermo Fisher Scientific, Waltham, MA, USA), 25mM Tris HCL Ph 7.5 (1185-53-1, Millipore Sigma, Burlington, MA, USA), 100mM NaCL (7647-14-5, Sigma-Aldrich), Halt protease inhibitor cocktail (78430, Thermo Fisher Scientific) and Phenylmethanesulfonyl Fluoride (329-98-6, Millipore Sigma). Samples were stored at − 80 °C and cytokine analysis was performed at the Human Immune Monitoring Core (Stanford University) as previous described [16]. Briefly, Mouse 48-plex Procarta kits (EPX480-20834-901, Thermo Fisher Scientific) were employed and plates were read using FM3D FlexMap instrument with a lower bound of 50 beads per sample per cytokine. Custom Assay Chex control beads were added to all samples. The median fluorescence intensity (MFI) was averaged over duplicate wells for each cytokine per sample on each plate. The MFI for sample media, serving as the background, was averaged and then adjusted by adding two standard deviations. The presented data represents the sample MFI minus the media MFI.

### Enzyme-linked immunosorbent assay (ELISA)

The cochleae were dissected and homogenized in the buffer provided by the kits. The tissues were then centrifuged at 18,000 x g for 20 min at 4 °C, and the resulting supernatant was used for the ELISA assay. The assay processes followed each manufacture’s instruction separately. The ELISA kits used included NLRP3 (Mouse NLRP3 ELISA Kit, ab279417, Abcam. Waltham, MA 02453 USA), IL-1β (Mouse IL-1 beta ELISA Kit, ab100705, Abcam), and IL-18 (Mouse IL-18 ELISA Kit, ab216165, Abcam). The optical density (OD) values were recorded using a TECAN microplate reader (Tecan spark, Austria).

### Quantitative real-time PCR (qPCR)

The inner ears were dissected out and the surrounding soft tissues were removed at 7d after inoculation. The inner ears were washed with fresh PBS more than 20 times to clean the surface of the cochleae, as described in a previous publication [6]. The cochleae were separated from vestibule in RLT buffer from the RNeasy mini kit (74004, Qiagen, Germantown, MD, USA). Samples were then mechanically homogenized, and RNA was extracted following manufacturer protocol. Reverse transcription to complementary DNA (cDNA) was performed using the SuperScript VILO cDNA Synthesis Kit (11754050, Thermo Fisher Scientific) according to manufacturer protocol. qPCR was performed using the CFX Maestro software on the CFX Connect Real-Time PCR System (Bio-Rad, Hercules, CA, USA). For qPCR reactions, 1 µl of 20 ng/µl cDNA were added to a 20 µl reaction using TaqMan Gene Expression Assays (Thermo Fisher Scientific) for NLRP3 (Mm00840904_m1), Pycard (Mm00445747_g1), Caspase-1 (Mm00438023_m1), IL-1β (Mm00434228_m1), IL-18 (Mm00434226_m1) and GAPDH (Mm99999915_g1). Samples were run in triplicate. mRNA relative expression was normalized to the house keeping gene GAPDH and calculated using the comparative CT method.

### Auditory measurements

Auditory brainstem responses (ABRs) and distortion product otoacoustic emissions (DPOAEs) were serially measured in a cohort of mice as previously described [24, 25]. Briefly, the ABR potentials were measured from needle electrodes positioned at the bottom of the tympanic bulla and at the vertex of the head, with a ground electrode placed in the rear leg. A bioamplifier (DP-311, Warner Instruments, Hamden, CT, USA) was used to amplify the signal 10,000 times.

The sound intensity level was raised in 10 dB steps from 10 to 80 dB SPL and the sound frequency was varied between 4 and 46 kHz. At each sound level, 260 responses were sampled and averaged. The peak-to-peak value of the ABR was measured and the threshold at each frequency was calculated to be when this value was three standard deviations above the noise floor. If no ABR response was detected, even at our equipment limits of 80 dB SPL, we arbitrarily defined the threshold to be 80 dB SPL.

DPOAEs were measured by a probe tip microphone (type 4182, Bru¨el & Kjaer, Denmark) in the external auditory canal. The frequency response of this microphone was measured using a free field microphone with a flat frequency response out to 100 kHz (type 4939, Bru¨el & Kjaer). This calibration curve was then used to adjust the DPOAE amplitudes we measured during the experiments. The sound stimuli for eliciting DPOAEs were two 1 s sine-wave tones of differing frequencies (F2 = 1.226F1). We varied the range of F2 from 4 to 46 kHz. The two tones were of equal intensities and stepped from 20 to 80 dB SPL in 10 dB increments. The amplitude of the cubic distortion product was measured at 2*F1–F2. The threshold at each frequency was calculated to be when the DPOAE was 0.5 dB SPL and two standard deviations above the noise floor. If no DPOAE response was detected, even at our equipment limits of 80 dB SPL, we arbitrarily defined the threshold to be 80 dB SPL.

### Statistical analysis

Statistics were performed using GraphPad Prism 9.0 (GraphPad Software Inc., La Jolla, CA, USA). All values in the figures are presented as mean ± standard deviation (SD) or standard error (SE). For three or more groups, we first performed an ANOVA analysis. For a P value < 0.05, we then used t-tests to compare pairs of subgroups. We compared data between groups using the unpaired, two-tailed Student’s t test. *P* < 0.05 were considered statistically significant. The mean ± SD/SEM and the P-values are calculated based on rounding the raw data to the nearest integer.

## Results

### Macrophage depletion does not affect cochlear function and immune homeostasis

The proliferation, differentiation, and survival of macrophages is regulated by colony-stimulating factor 1 receptor (CSF1R) signaling [26]. CSF1R signaling can be inhibited using the CSF1R inhibitor PLX5622. Administration of PLX5622 through rodent chow depletes approximately 90% of microglia from the adult brain and spinal cord without causing functional impairment [27–33]. PLX5622 has also previously been shown to successfully deplete ~ 94% of cochlear resident macrophages [34]. We used it to deplete cochlear resident macrophages in the current study and counted macrophages within the line showing in Fig. S1A. After feeding mice with PLX5622 diet chow for 14 days (14d), ~ 93±4% of resident macrophages were depleted compared to the control group fed a normal diet (Fig. S1. Unpaired t-test, *p* < 0.001). Subsequently, we examined the cochlear cytokines using a Luminex Bead Array and found no changes in the macrophage depletion group, compared to control (Fig. S2). Furthermore, we conducted functional tests of hearing by measuring the auditory brainstem response (ABR) and Distortion Product Otoacoustic Emissions (DPOAE), both of which showed no hearing changes in the PLX5622 group compared to the control group (Fig. S3A-B). Lastly, we counted Outer Hair Cells (OHCs) number in the cochlear base, middle and apex (Fig. S4C-E, S4F-H). No OHC loss was observed in either group. Taken together, these data demonstrated that the depletion of cochlear resident macrophages does not affect cochlear function and cochlear immune homeostasis.

### The NLRP3 knockout does not affect cochlear function

We also performed ABR & DPOAE tests to assess the hearing baseline of NLRP3^−/−^ mouse. The data revealed no significant difference in hearing between the NLRP3^−/−^ group and the control group (Fig. S4).

### The NLRP3 knockout is confirmed in the cochlear tissue

To confirm that the NLRP3 protein was completely knocked out in the cochlear tissue, we performed ELISA. The data revealed that NLRP3 was not detectable in NLRP3^−/−^ and NLRP3^−/−^ CSOM mice (Fig. S5).

### Macrophage depletion or NLRP3^−/−^ does not alter the middle ear CSOM infection

Prior to investigating how the depletion of cochlear resident macrophages or NLRP3 knockout condition affects the inner ear, we assessed whether these conditions affect the middle ear infection. A change in the overall middle ear infection burden could alter the inner ear response. Immediately after cessation of the PLX5622 diet chow, 5 µl of PAO1 persister cells at a concentration of 3.7 × 10^6 colony forming unit (CFU)/ml were inoculated into the middle ear cavity. An equivalent amount of PAO1 was also inoculated into NLRP3^−/−^ mice. We then assessed the disease development in the middle ear at 7 days (7d) post infection. CFU assays were performed (Fig. S6). The average CFU/mg middle ear were 167 ± 78 in control CSOM, 160 ± 76 in PLX5622 CSOM, and 132 ± 29 in NLRP3^−/−^ CSOM, respectively. There was no significant difference among the three groups: control CSOM, PLX5622 group and NLRP3^−/−^ group (Excel One-Way ANOVA, F = 0.7, *P* = 0.5). The CFU range and variability we found in these groups was also similar to our original published model [5]. The data revealed that neither macrophage depletion nor NLRP3 deletion significantly affects the development and burden of the CSOM middle ear infection.

### Cochlear macrophages are reduced in the PLX5622 group following middle ear infection

**Fig. 1:**
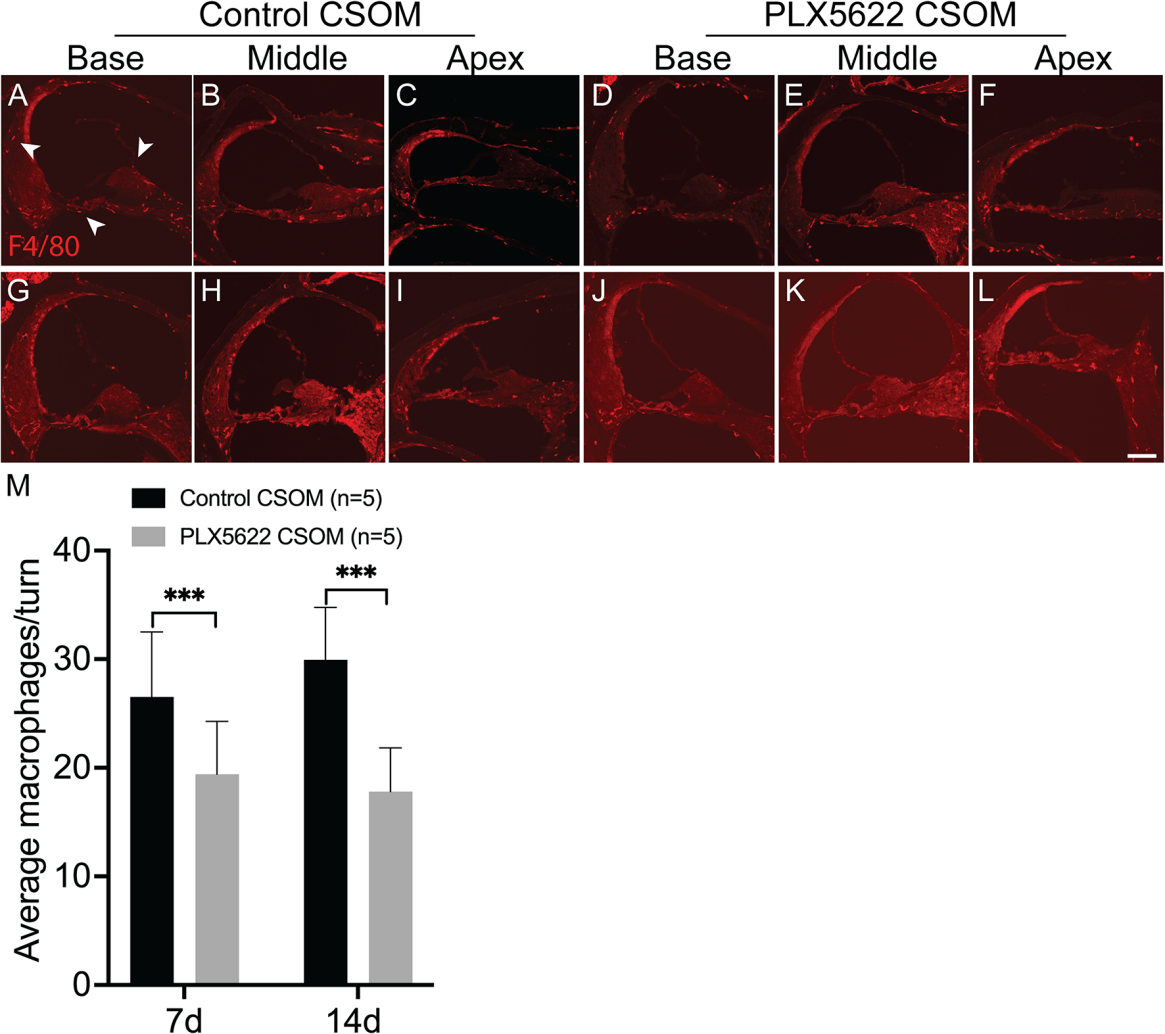
Macrophages are reduced following PLX5622 treatment in CSOM. Cryosections of the cochlea (basal, middle and apical turn) are shown in control (left) and PLX5622 treated (right) CSOM mice at different timepoints: 7d (**A**-**F**) and 14d (**G**-**L**). Panel M shows a significant decrease in the number of macrophages per turn at 7d (*p* ≤ 0.001) and 14d (*p* < 0.001) in the PLX5622 treated group. The number of mice per group is shown in parentheses. The data represents mean +/- SD. Red = F4/80 (arrows). Scale bar = 100 μm

After confirming the middle ear infection burden was not different between groups, cochlear macrophages were counted as described in Fig. S1A at 7d and 14d after middle ear infection. Macrophages were observed in the stria vascularis, spiral ligament, spiral ganglion neurons, beneath the basilar membrane in the scala tympani and Reissner’s membrane, similar to previously described (Fig. 1A, arrows). The mean number of cochlear macrophages per turn was 26.5 ± 6.0 at 7d (Fig. 1A-C) and 29.9 ± 4.8 at 14d (Fig. 1G-I), respectively, in the control group. In contrast, it was19 ± 4.9 at 7d (Fig. 1D-F) and 18 ± 4.0 at 14d (Fig. 1J-L), respectively, in the PLX5622 group, The macrophages in the PLX5622 group showed a significant reduction compared to the control group at 7d (Fig. 1M. Unpaired t-test, *p* ≤ 0.001) and 14d (Fig. 1M. Unpaired t-test, *p* < 0.001), respectively, even though PLX5622 treatment was ceased at the time of the middle ear infection creation.

### OHCs have a higher survival rate in the PLX5622 treated CSOM group

**Fig. 2:**
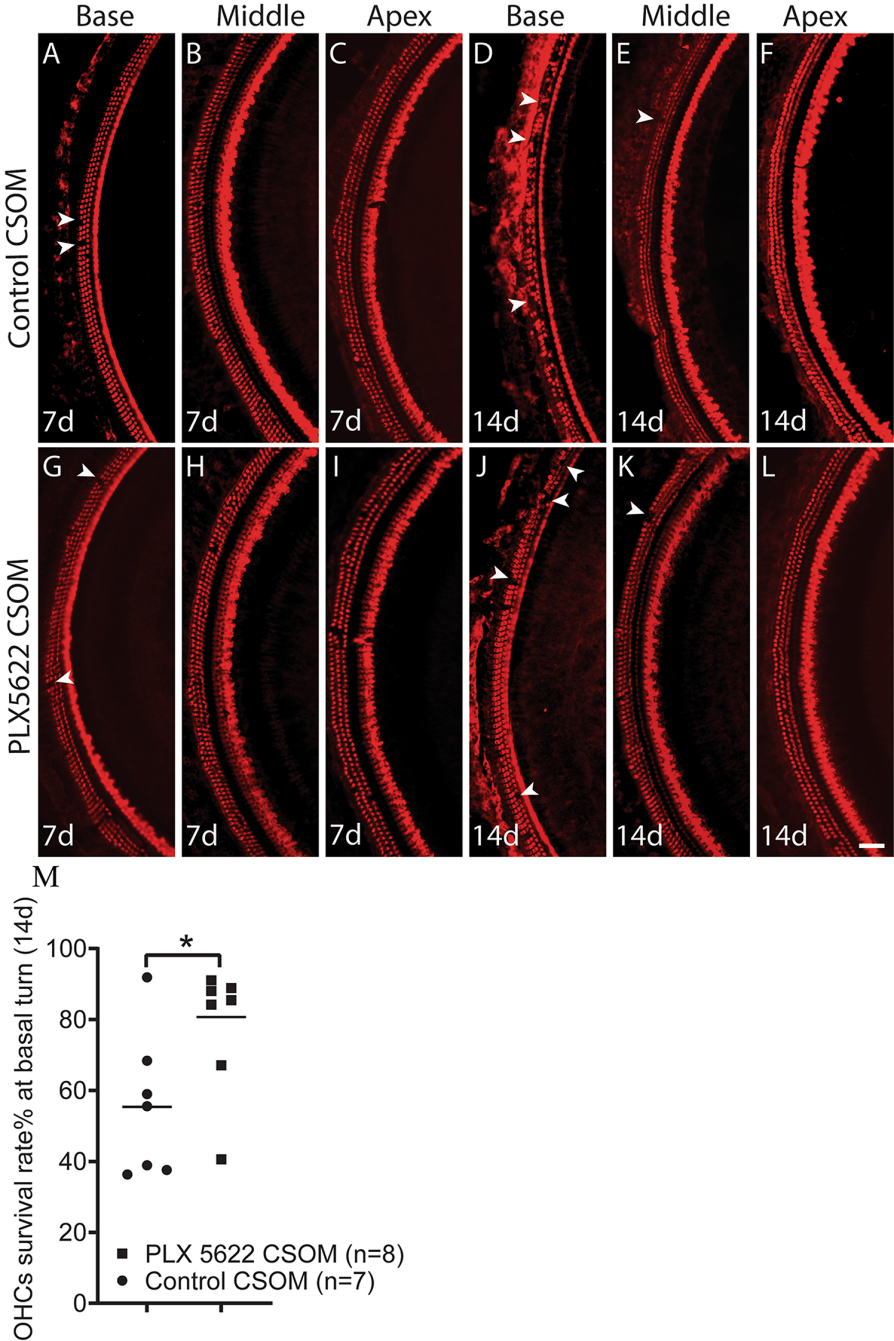
Depletion of cochlear macrophages in CSOM improves OHC survival. Wholemount samples of the cochlea (basal, middle, and apical turn) are shown in control (top row) and PLX5622 treated (bottom row) mice in CSOM at 7d (**A**-**C** and **G**-**I**) and 14d (**D**-**F** and **J**-**L**). (**M**) The OHC survival rate in % was compared to OHC in healthy cochlear basal turn at 14d. The OHC survival rate was significantly higher in PLX5622 treated CSOM compared to control CSOM. The number of mice per group is shown in Parentheses. The data represents mean +/- SD. Red = Myosin VIIa. Arrows show HC loss. Scale bar = 100 μm

We evaluated the effect of macrophage depletion, via PLX5622 treatment, on OHC survival in CSOM. Slight loss of OHCs was observed in the cochlear base at 7d in the control CSOM group (Fig. 2A), as well as in the PLX5622 CSOM group (Fig. 2G). No OHC loss was observed in the middle and apex in both the control CSOM (Fig. 2B-C) and PLX5622 CSOM groups (Fig. 2H-I). At 14d, minimal loss of OHCs was also found in the cochlear middle turns in both the control CSOM (Fig. 2E, arrow) and PLX5622 CSOM (Fig. 2K, arrow), but no OHC loss was observed in the cochlear apex in both groups (Fig. 2F and L). Notably, the OHC survival rate was 80.7 ± 18.7% in PLX5622 CSOM (Fig. 2J, arrows), compared to 55.4 ± 20.3% in the cochlear base of control CSOM (Fig. 2D, arrows). This difference in the OHC survival rate was statistically significant (Fig. 2M, Unpaired t-test, *p* = 0.026), indicating that reducing the macrophage number in CSOM is protective for OHCs.

### NLRP3 inflammasome and its downstream cytokines are upregulated in CSOM

**Fig. 3:**
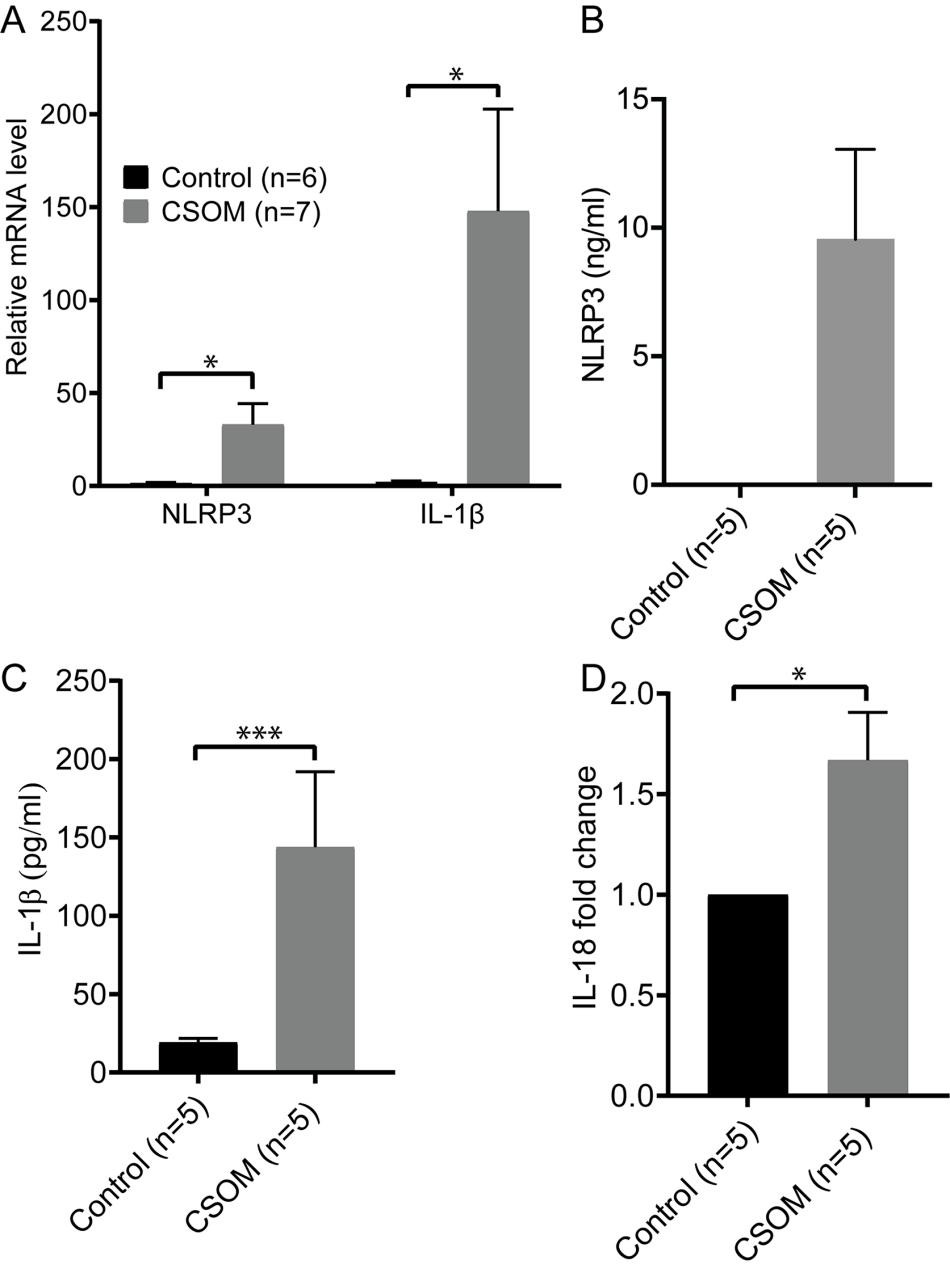
NLRP3, IL-1β and IL-18 are increased in the CSOM cochlea. **A** shows that mRNA expression of NLRP3 (*p* = 0.018) and IL-1β (*p* = 0.032) were significantly elevated in CSOM cochleae compared to control cochleae at 7d. mRNA data is shown as mean+/- SEM. Protein levels of NLRP3, IL-1β and IL-18 were determined in control and CSOM cochleae at 7d using ELISA. **B** shows a significant increase of NLRP3 in CSOM, whereas it was undetectable in the control group. **C** & **D** show a significant increase of IL-1β in CSOM (*p* < 0.001) and IL-18 in CSOM (*p* = 0.013). The number of mice is shown in parentheses. ELISA data is shown as mean +/- SD

The NLRP3 inflammasome activation in immune cells results in the secretion of the pro-inflammatory cytokines IL-1β and IL-18, mediated by caspase-1. We assessed whether the productions of the NLRP3 inflammasome and its downstream cytokines are upregulated in CSOM. Quantitative RT-PCR was conducted to assess the mRNA levels of NLRP3 and IL-1β. Their mRNA expression was found to be significantly elevated in CSOM (42.1 ± 13.3 for NLRP3, 199.2 ± 73.4 for IL-1β) compared to control (non-CSOM) cochleae (1.91 ± 0.91, *p* = 0.018 for NLRP3; 2.83 ± 1.55, *p* = 0.032 for IL-1β) at 7 days following middle ear infection (Fig. 3A). ELISA assays were then performed to test the protein levels of NLRP3 and its downstream factors at 7d after middle ear infection. The NLRP3 level was detected in the CSOM group (9.56 ± 3.5 ng/ml) compared to the control group, where it was undetectable (Fig. 3B). Significant changes of IL-1β levels were observed in the CSOM group (143.7 ± 48 pg/ml) compared to the control group (19.2 ± 2.5 pg/ml, Unpaired t-test, *p* < 0.001. Figure 3C). IL-18 protein exhibited a nearly 1.67 ± 0.24-fold increase in CSOM group (Unpaired t-test, *p* = 0.013. Figure 3D). The upregulation of NLRP3, IL-1β and IL-18 confirms the NLRP3 inflammasome activation in CSOM.

### NLRP3 complex factors and its downstream cytokines are downregulated in PLX5622 CSOM

**Fig. 4:**
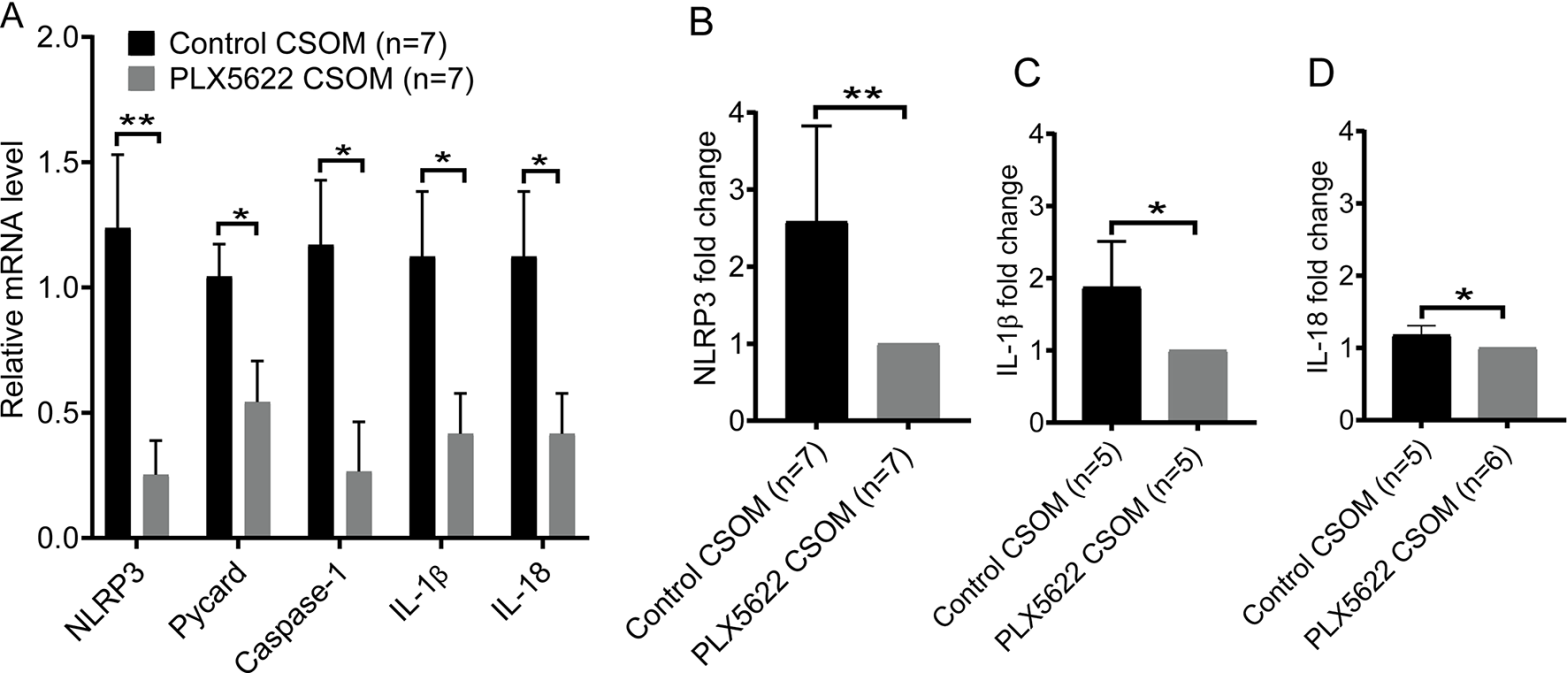
The mRNA and protein levels of NLRP3 inflammasome and its downstream cytokines decrease when macrophages are depleted in the cochlea. **A**: The relative mRNA levels of NLRP3, Pycard, Caspase-1, IL-1β, and IL-18 were determined in cochleae using qRT-PCR in both control (black) and PLX5622-treated (grey) mice at 7d in CSOM. A significant decrease in mRNA levels was observed for all these genes following the depletion of cochlear macrophages (NLRP3: *p* = 0.01, Pycard: *p* = 0.033, Caspase-1: *p* = 0.017, IL-1β: *p* = 0.04, and IL-18: *p* = 0.012). **B**: NLRP3 showed a significant fold decrease in the PLX5622 treatment group (*p* = 0.008). **C**: IL-1β showed a significant fold decrease in the PLX5622 treatment group (*p* = 0.024). **D**. IL-18 showed a significant fold decrease in the PLX5622 treatment group (*p* = 0.027). The number of mice per group is shown in parentheses. Data are shown as mean ± SEM in qPCR and as mean ± SD in ELISA

To assess if the NLRP3 inflammasome activation is suppressed when macrophages are reduced in CSOM, we measured the mRNA level of NRLP3 complex factors including NLRP3, Pycard, Caspase-1 and its downstream cytokine IL-1β and IL-18 using qRT-PCR. GAPDH was used as internal reference. Data was analyzed in mice at 7d following middle ear infection in both the control CSOM and PLX5622 CSOM (Fig. 4). The relative mRNA expression was lower in PLX5622 CSOM (0.25 ± 0.14) than in control CSOM (1.24 ± 0.29) for NLRP3 (*p* = 0.01), in PLX5622 CSOM (0.54 ± 0.16) than in control CSOM (1.04 ± 0.13) for Pycard (*p* = 0.033), in PLX5622 CSOM (0.27 ± 0.2) than in control CSOM (1.17 ± 0.26) for Caspase-1 (*p* = 0.017), in PLX5622 CSOM (0.42 ± 0.16) than in control CSOM (1.12 ± 0.26) for IL-1β (*p* = 0.04), and in PLX5622 CSOM (0.39 ± 0.17) than in control CSOM (1.1 ± 0.17) for IL-18 (*p* = 0.012). Furthermore, we measured the protein level of the NLRP3 inflammasome using ELISA assays at 7d after middle ear infection. The control CSOM group exhibited a 2.58 ± 1.24-fold increase in NLRP3 (*p* = 0.008), a 1.87 ± 0.64-fold increase in IL-1β (*p* = 0.024) and a 1.18 ± 0.13-fold increase in IL-18 (*p* = 0.027). The statistical analysis was performed with an unpaired t-test, respectively. Taken together, the qPCR and ELISA data confirm the upregulation of NLRP3, IL-1β, and IL-18, indicating NLRP3 inflammasome activation in CSOM. The data reveal that the downregulation of the activated NLRP3 complex factor correlates with the reduced number of macrophages in the PLX5622 CSOM group.

### The number of cochlear macrophages is unaffected in NLRP3^−/−^ CSOM

**Fig. 5:**
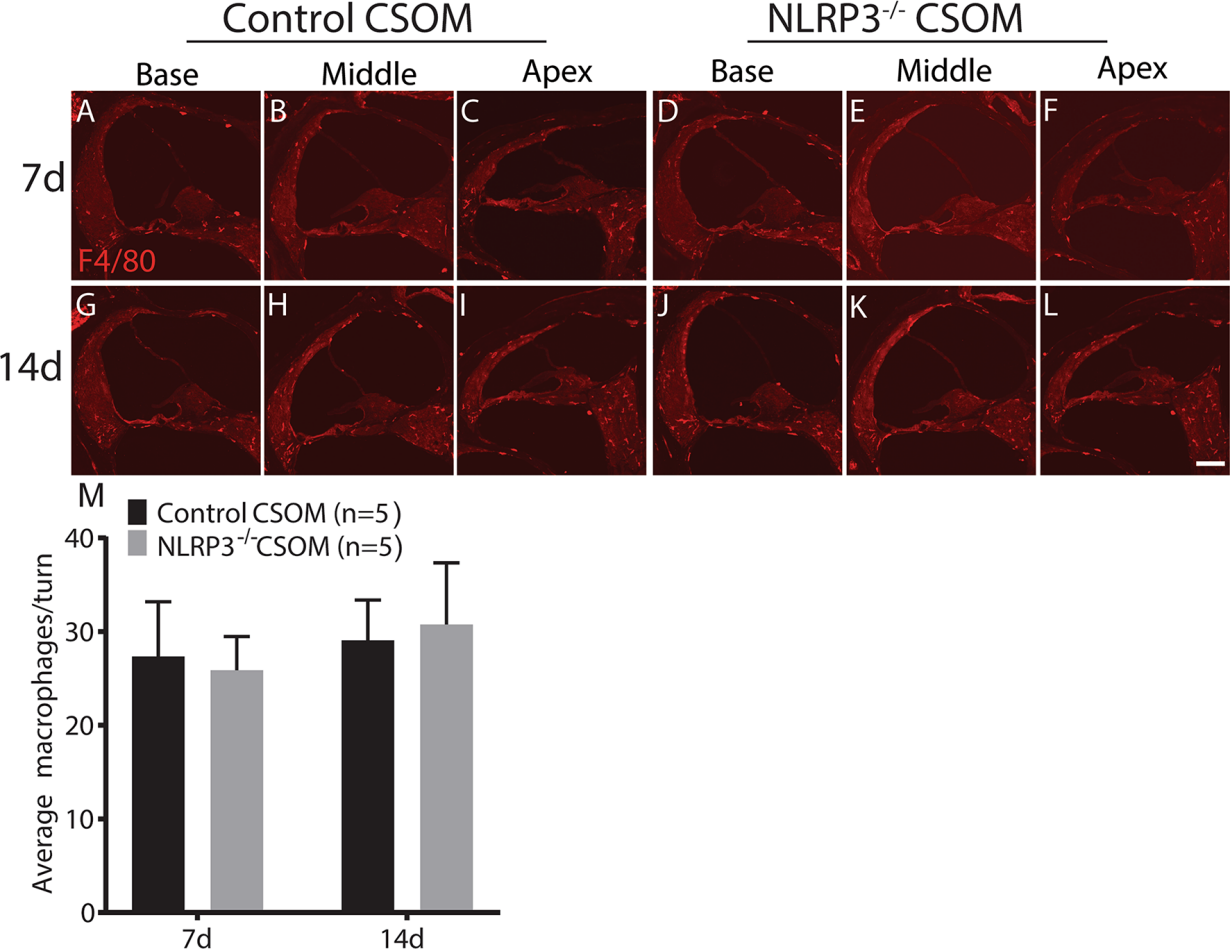
NLRP3^−/−^ does not affect the number of macrophages in the cochlea in CSOM. Cryosections of the cochlea (basal, middle and apical turn) are shown in control (left) and NLRP3^−/−^ (right) mice at 7d (**A**-**F**) and 14d (**G**-**L**) in CSOM. Panel M shows the average number of macrophages per turn in control and NLRP3^−/−^ mice at 7d and 14d in CSOM. The number of mice per group is shown in parentheses. Data is shown as mean +/- SD. Red = F4/80. Scale bar = 100 μm

To evaluate the contribution of the NLRP3 inflammasome within macrophages to OHC loss in CSOM, we used the NLRP3 ^−/−^ mouse model, where the NLRP3 inflammasome has been validated as not functional [23]. We first assessed if the number of macrophages in the cochlea was affected in the NLRP3^−/−^ CSOM. We created CSOM, as described above, and counted macrophages in the NLRP3^−/−^ mouse cohort, The macrophage numbers remained unchanged in NLRP3^−/−^ CSOM (31.04 ± 9.04) compared to control CSOM (30.4 ± 7.5) at 7d (Fig. 5A-F and M. Unpaired t-test, *p* = 0.8) and in NLRP3^−/−^ CSOM (36.5 ± 9.4) compared to control CSOM (32.75 ± 6.6) at 14d (Fig. 5G-M. Unpaired t-test, *p* = 0.1). The data demonstrates that NLRP3 deletion does not affect the overall macrophage number, including both resident and migrating macrophages.

### OHCs are protected in NLRP3^−/−^ CSOM

**Fig. 6:**
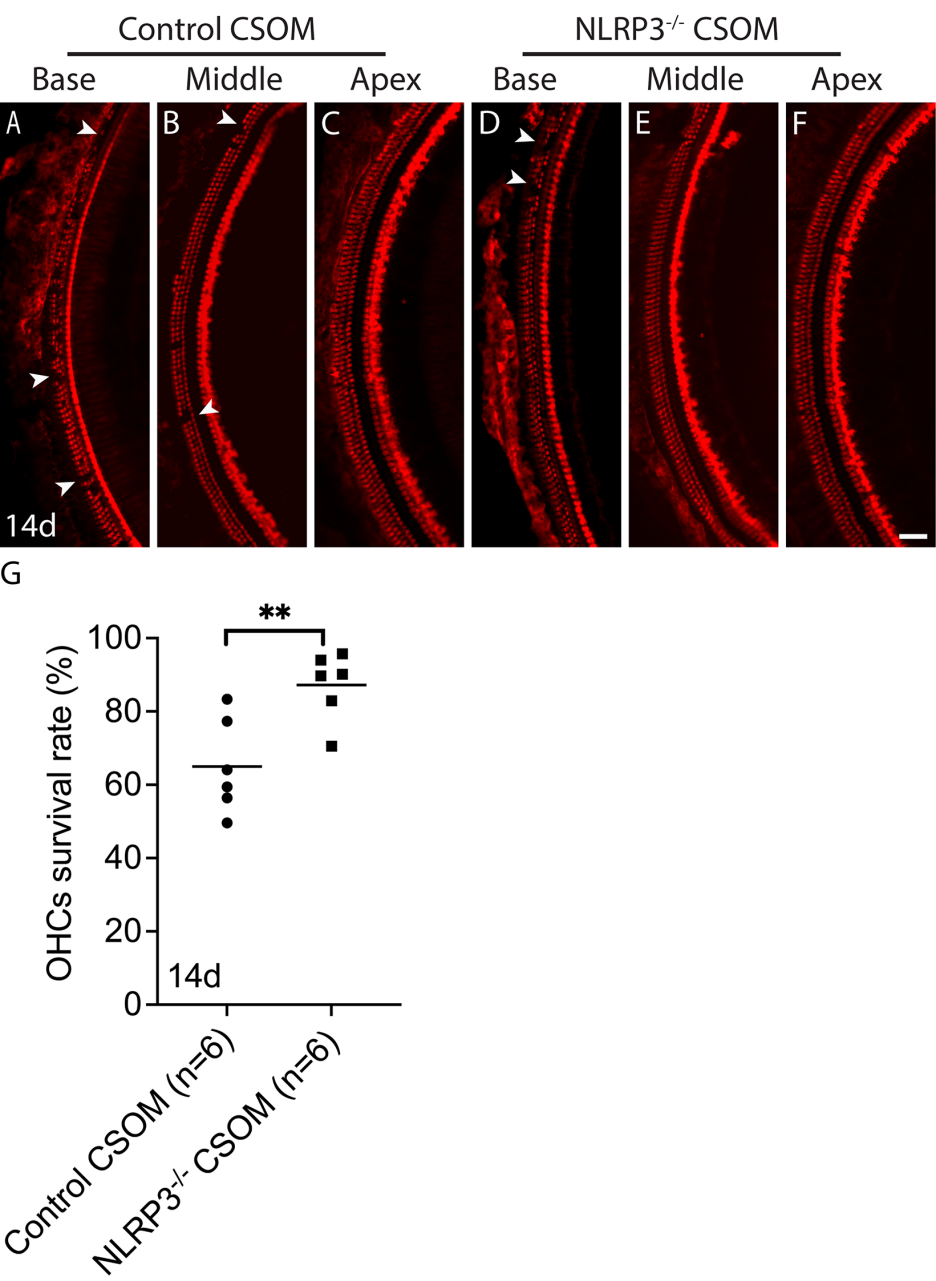
An active NLRP3 inflammasome leads to OHC loss in CSOM. Representative wholemount sections of the cochlea (basal, middle and apical turn) in CSOM at 14d from control (**A**-**C**) and NLRP3^−/−^ (**D**-**F**) CSOM mice. Panel G shows the OHC survival rate in % in the cochlear basal turn of both groups, NLRP3^−/−^ is protective for OHC loss in CSOM (*p* = 0.007). Red = Myosin VIIa. The number of mice per group is shown in parentheses. Data is shown as mean +/- SD. Arrows show HC loss. Scale bar = 100 μm

Next, we counted surviving OHC at 14d (Fig. 6). ~65 ± 13% of OHCs survived at the cochlear basal turn, the high frequency region, in the control CSOM group (Fig. 6A). In contrast, ~ 87 ± 9.3% of OHCs survived at the cochlear basal turn in NLRP3^−/−^ CSOM group (Fig. 6D). The OHC survival rate is greater in the NLRP3^−/−^ CSOM group (Fig. 6G. Unpaired t-test, *p* = 0.007). In addition, little loss of OHCs was observed in the middle turn of the control CSOM group (Fig. 6B. Arrows), but not in the middle turn of the NLRP3^−/−^ CSOM group. No OHC loss occurred in the apex in both groups (Fig. 6C and F). The data indicates that the OHC protection resulted from the NLRP3 deletion rather than a change in macrophage number in the NLRP3^−/−^ CSOM mouse.

### Inhibiting the NLRP3 inflammasome activation and IL-1receptor prevents OHC loss

As NLRP3 deletion preserves OHCs in NLRP3^−/−^ CSOM mice, we aimed to see if this pathway could be therapeutically targeted to prevent OHC loss in CSOM mice. The diarylsulfonylurea compound MCC950, has been shown to inhibit the NLRP3 oligomerization and active confirmation by directly targeting the NLRP3 ATP-hydrolysis motif [35] and reduce IL-1β production. MCC950 treatment has been shown to rescue neonatal lethality in a mouse model of cryopyrin-associated periodic syndromes (CAPS) and alleviate hearing loss [36, 37].

**Fig. 7:**
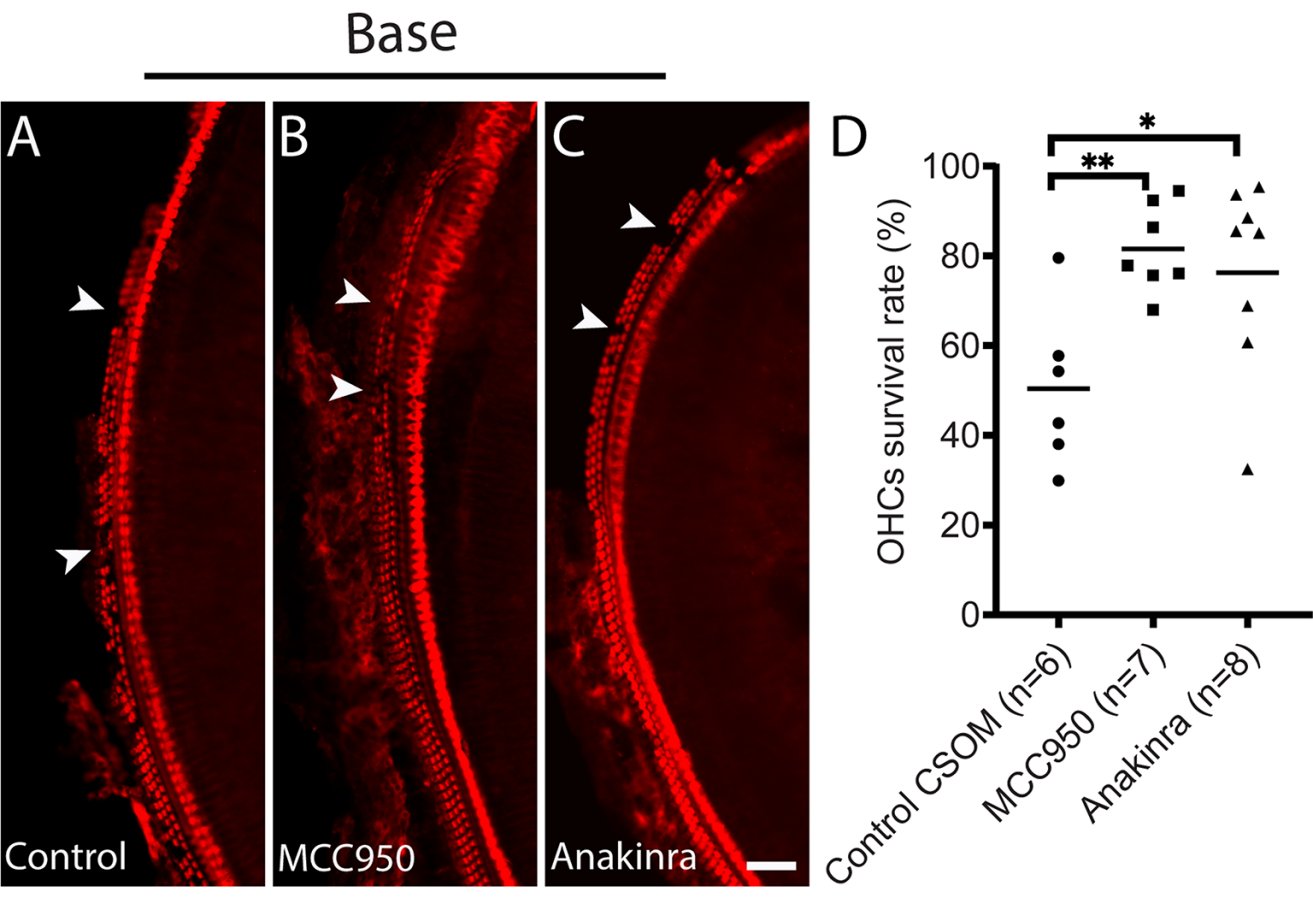
Targeting the NLRP3 inflammasome activation and IL-1β receptor protects OHC loss in CSOM. Representative wholemount sections of the cochlear basal turn (**A**-**C**) are shown at 14d in CSOM. A represents the control, without treatment, **B** and **C** were treated daily with either the NLRP3 inhibitor MCC950 i.p. (**B**) or the IL-1 receptor antagonist Anakinra i.p. (**C**). D shows the quantification of the OHC survival rate in % in all groups at 14d in CSOM (*p* = 0.002 between MCC950 and control CSOM, *p* = 0.03 between Anakinra and control CSOM). The number of mice per group is shown in parentheses. Data is shown as mean +/- SD. Red = Myosin VIIa. Arrows show HC loss. Scale bar = 100 μm

Anakinra is a recombinant form of the human interleukin-1 receptor antagonist (IL-1Ra), structurally similar to endogenous IL-1Ra. It functions by competitively binding to the interleukin-1 (IL-1) receptor, inhibiting the biological activities of IL-1α and IL-1β. This anti-inflammatory action makes Anakinra effective in treating conditions associated with excessive IL-1 activity, such as rheumatoid arthritis for which it has already been approved for. Anakinra has been used to treat the patients with hearing loss in CAPS [38]. In the current study, intraperitoneal injections of both MCC950 and Anakinra were administered daily, respectively, after creating the middle ear infection. The OHC survival rate in the cochlear base was ~ 50.4 ± 17.6% in the control CSOM group, ~ 81.5 ± 9.7% in the MCC950 treatment group, and ~ 76.2 ± 21.3% (Fig. 7A-C. arrows) in the Anakinra treatment group. There was a significant difference between the MCC950 treatment and control CSOM (Fig. 7D. Unpaired t-test, *p* = 0.002), and also between Anakinra and control CSOM (Fig. 7D. Unpaired t-test, *p* = 0.03). The data reveals that inhibiting the NLRP3 inflammasome with MCC950 and administration of the IL-1 receptor antagonist Anakinra prevent OHC loss in CSOM, and therefore shows an ability to cross the blood labyrinthine barrier. Finally, we compared the OHC survival rate among the control CSOM groups, indicating no significant differences (Fig. S7. Excel One-Way ANOVA. *P* = 0.35, F = 1.1).

This suggests the reliability of the CSOM mouse model. We also compared the OHC survival rate among the PXL5622 CSOM, NLRP3^−/−^ CSOM, MCC950 treatment, and Anakinra treatment groups, demonstrating no significant differences (Fig. S7). Excel One-Way ANOVA. *P* = 0.67, F = 0.51). The data verify a direct relationship between NLRP3 inflammasome activation and cochlear macrophages, and no treatment is superior to the others.

## Discussion

CSOM can lead to irreversible SNHL for which there is no current prevention or treatment [39–41]. Our group developed and validated an animal model that replicates human CSOM [5]. In this model, induced by a pathogen in the middle ear, macrophages were identified as the major immune cell associated with OHC loss in the cochlea [6], but it was unknown if the macrophages were causative for the SNHL. In the current study, we investigated the mechanism underlying cochlear macrophage related hearing loss in the CSOM mouse model and uncovered a novel immune mediated mechanism for inner ear injury in infectious ear diseases.

The immune response of the inner ear has been broadly studied in various mouse models and macrophages have been shown to play both protective and damaging roles. In the context of noise induced hearing loss (NIHL) [9, 12, 13], where monocytes/macrophages migrate into the cochleae and serve a phagocytic function following otoacoustic trauma, they provide an advantage for repair. Manickam et al. found that in the absence of macrophages, depleted using the CSF1R inhibitor PLX5622, repair does not occur to the synapses following NIHL [34]. Another research group found that the depletion of cochlear macrophages using the CSF1R inhibitor PLX3397 reduced platinum accumulation in the cochlea, suggesting macrophage phagocytosis may lead to its accumulation associated toxicity [14]. Notably, these studies were conducted in models without a pathogen as the stimulus for injury. We demonstrated significant survival of OHCs in the cochlea when macrophages were depleted, indicating they have an overall harmful effect on the hair cell in CSOM, in contrast to the above-described protective role.

The NLRP3 inflammasome activation is involved in the development of different diseases, including metabolic disorders, autoinflammatory syndromes, gout disease, atherosclerosis, and Alzheimer’s disease [42–46]. In a mouse model with a gain-of-function mutation of NLRP3, the NLRP3 inflammasome activation by systemic LPS injection leads to cochlear inflammation and SNHL, hinting at the role of the NLRP3 inflammasome in the ear [37]. Gain-of-function mutations of NLRP3 are known to cause syndromic hearing loss in systemic autoinflammatory diseases- CAPS and autosomal dominant non-syndromic hearing loss locus (DFNA43) in patients [38, 47]. The genetics involved in CSOM are unknown, but the over 300 million people affected worldwide are unlikely to have gain-of-function mutations; instead, they are likely to have normally functioning NLRP3 inflammasomes. In the current study, NLRP3 and its downstream cytokines including IL-1β and IL-18 are elevated, indicating the NLRP3 inflammasome activation in CSOM. The expression of both mRNA and protein for NLRP3, Pycard, Caspase-1, IL-1β and IL-18 is downregulated when macrophages are depleted in CSOM, confirming that the NLRP3 inflammasome activation occurs in the cochlear macrophages. Furthermore, the survival rate of OHCs is higher in NLRP3^−/−^ and macrophage-depleted CSOM groups, confirming that the NLRP3 inflammasome activation within macrophages is the cause of OHC loss in CSOM. This finding has significant implications, as it demonstrates that a pathogen can trigger a normally functioning NLRP3 inflammasome, leading to pathology in the inner ear. IL-1β induces the expression of genes regulating fever, pain threshold, vasodilation, and hypotension. Its receptor prompts an endothelial cell response, facilitating the infiltration of immune cells into infected or damaged tissues [48, 49]. Anakinra, a recombinant human interleukin-1 (IL-1) receptor antagonist (IL-1Ra), has been widely used for the treatment of various diseases, specifically rheumatoid arthritis and CAPS [50]. MCC950 is a direct inhibitor of the NLRP3 inflammasome [35, 51]. MCC950 was developed as a therapeutic to be used in rheumatoid arthritis, however its progression in clinical development has stalled due to liver toxicity limitations [52]. In the current study, MCC950 and Anakinra administration started daily on day 2 after creating the CSOM model at day 0 and is continued until day 12, respectively. The survival rate of OHCs is significantly higher in both the MCC950 treatment and Anakinra treatment groups, indicating that OHCs are protected with these treatments.

The limitations of our study include the restriction of our scope to a 14-day period post middle ear infection. While we focused on OHCs, which are the most vulnerable cell type in the cochlea, it’s crucial to acknowledge that other cells within the cochlea may sustain damage beyond this timeframe, requiring further investigation. We generated the CSOM mouse model and used existing drugs to treat SNHL in CSOM. The translation of these findings to clinical applications in humans needs to address the difference in immune responses and efficacy. Despite these limitations, our study provides a potential target for preventing hearing loss in CSOM within a clinical context.

## Conclusions

Our study demonstrates that the NLRP3 inflammasome and its associated cytokines are upregulated in CSOM and downregulated, when macrophages are depleted in CSOM. Notably, OHCs are protected when macrophages are depleted and when the NLRP3 inflammasome is deleted in CSOM. Furthermore, OHC loss is demonstrably prevented with an inhibitor targeting NLRP3 and an IL-1 receptor antagonist. These findings strongly suggest that the NLRP3 inflammasome activation in cochlear macrophages contributes to OHC loss in CSOM, and targeting the NLRP3 inflammasome can prevent this loss.

## Electronic supplementary material

Below is the link to the electronic supplementary material.

Supplementary Material 1

## Data Availability

All data generated or analysed during this study are included in this published article [and its supplementary information files].

## Abbreviations

CSOM: Chronic suppurative otitis media
PA: Pseudomonas Aeruginosa
PCs: PA persister cells
SNHL: sensorineural hearing loss
OHC: outer hair cell
IHC: inner hair cell
NIHL: noise-induced hearing loss
IL-1β: interleukin-1β
IL-18: interleukin-18
