# Supplementary material for: Targeting the NLRP3 inflammasome in cochlear macrophages protects against hearing loss in chronic suppurative otitis media
Source: J Neuroinflammation. 2024 Sep 14;21:223. doi: 10.1186/s12974-024-03212-6 (PMC11402200; doi:10.1186/s12974-024-03212-6)
Supplement: Supplementary file 1 — Supplementary Material 1 [file 12974_2024_3212_MOESM1_ESM.docx]

**Supplementary Materials for**

**Targeting the NLRP3 inflammasome in cochlear macrophages protects against hearing loss in Chronic Suppurative Otitis Media**

Schiel et al.

Correspondence: apxia@stanford.edu; petersantamaria@stanford.edu


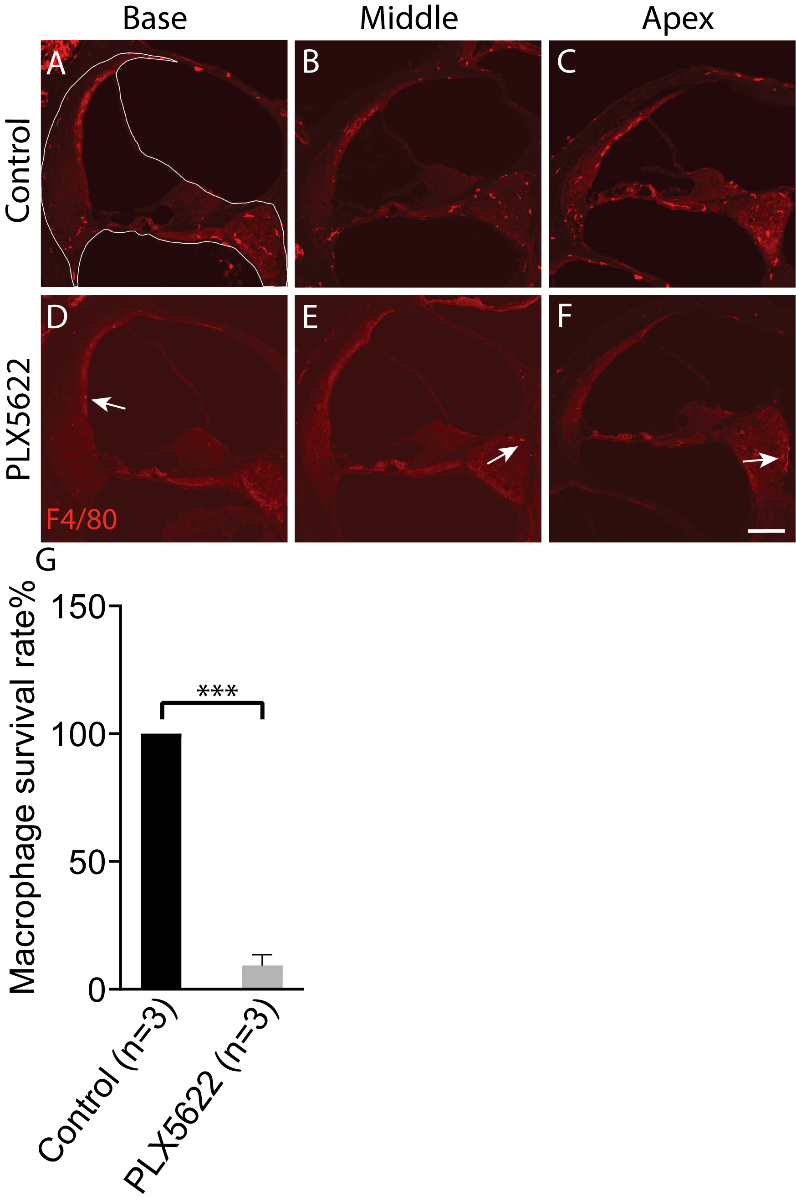


**Fig. S1. PLX5622 successfully depletes cochlear macrophages**

Cryosections of the cochlea (basal, middle and apical turn) are shown in control (A-C) and PLX5622 treated (D-F) mice after 14 days treatment. Panel G shows a significant decrease of macrophage survival rate in PLX5622 treated mice (p<0.001). The number of mice per group is shown in parentheses. The data represents mean +/- SD. Red = F4/80. Arrows point at cochlear macrophages (D-F). Scale bar = 100 μm.


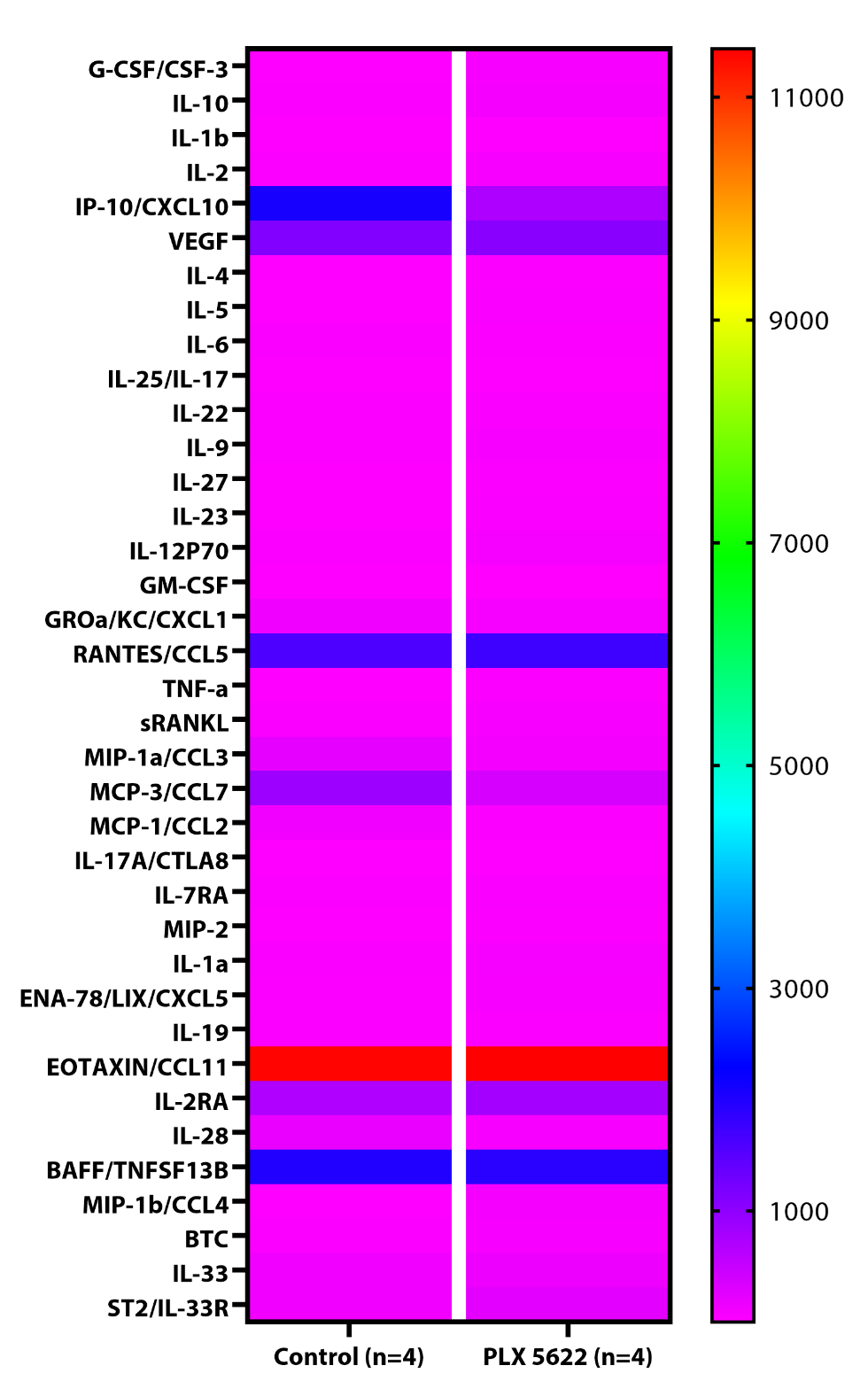


**Fig. S2. PLX5622 does not change the cytokine profile in the cochleae.**

A broad range of cytokines were measured in control (left) and PLX5622 treated (right) murine cochleae with Luminex Bead Array and were represented as a heatmap. There were no significant differences in cytokines between both groups. The number of mice per group is shown in parentheses.


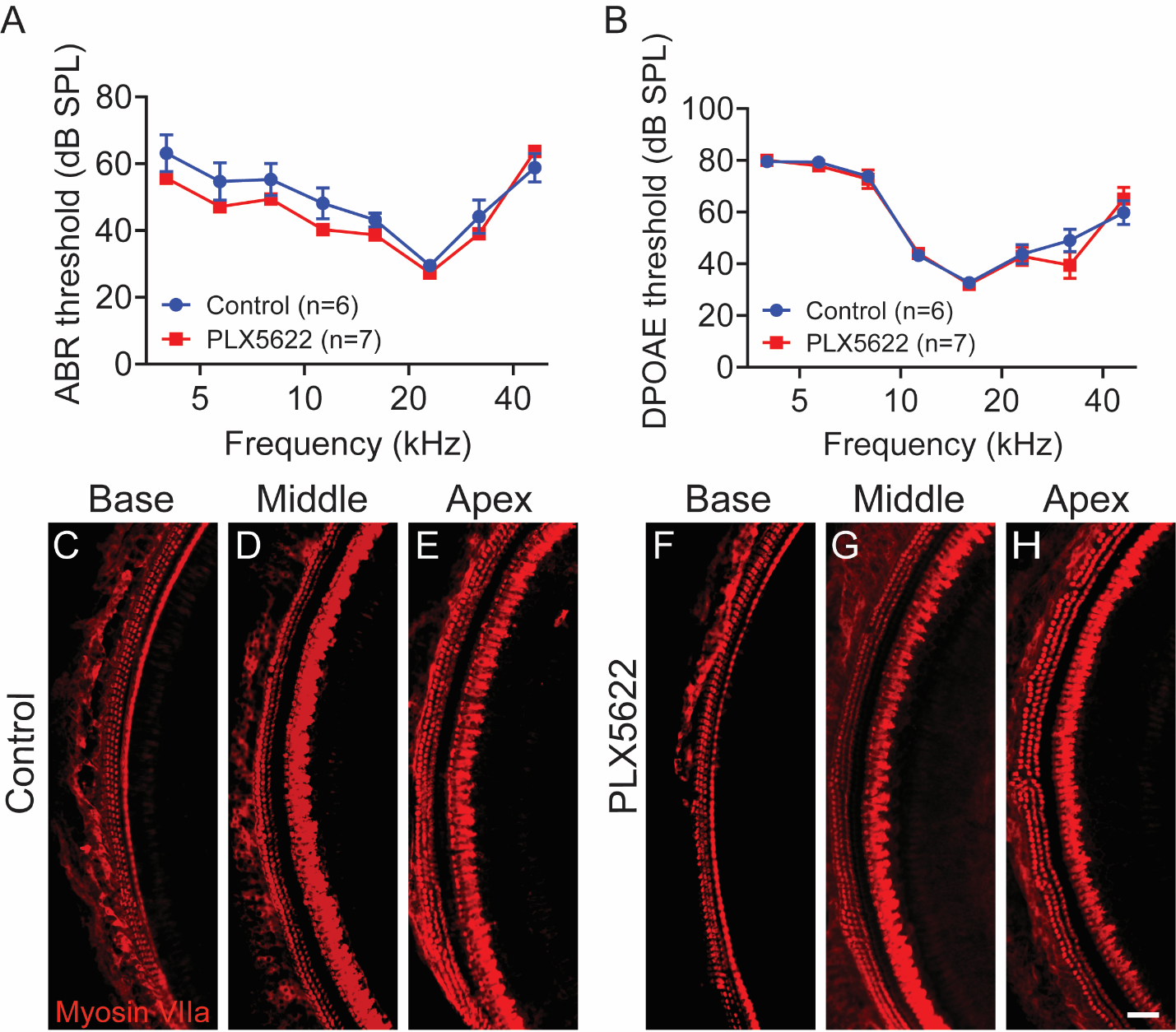


**Fig. S3. Depletion of cochlear macrophages does not affect hearing and HCs.**

(A) ABR and (B) DPOAE thresholds were measured in control (blue) and PLX5622 treated (red) mice. Wholemount sections of the cochlea show regular hair cells in all cochlear turns (base, middle, apex) in control (C-E) and PLX5622 treated (F-H) murine cochleae. The number of mice per group is shown in parentheses. The data represents mean +/- SD. Red = Myosin VIIa. Scale bar = 100 μm.


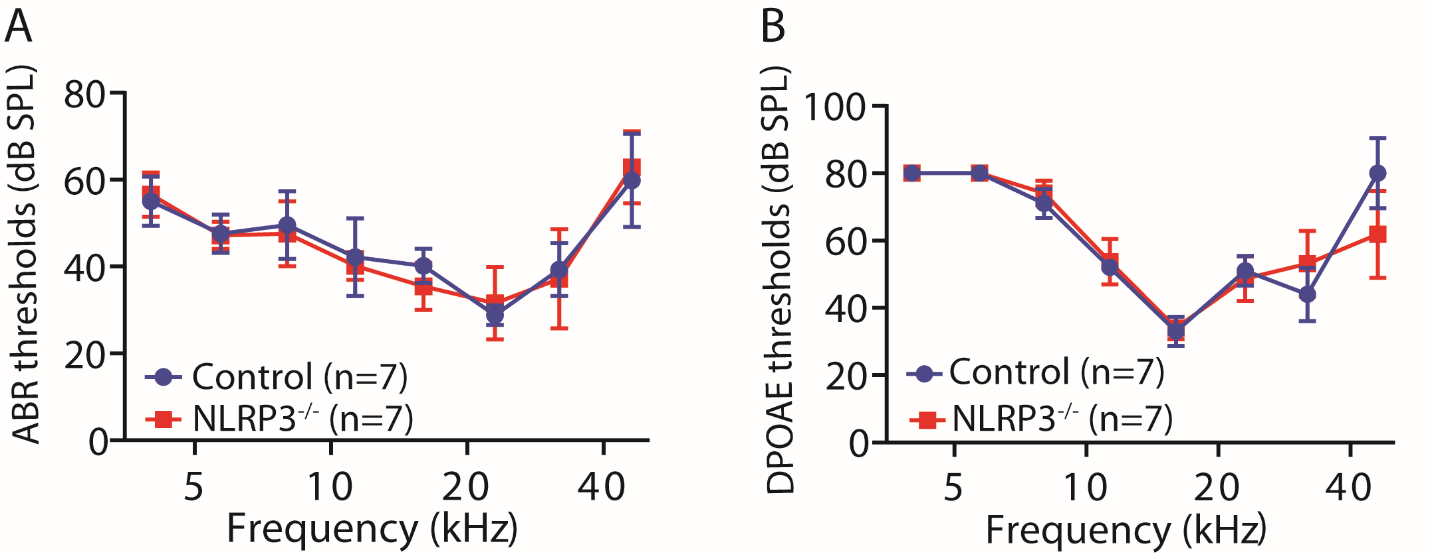


**Fig. S4. NLRP3 deletion does not affect hearing.**

(A) ABR and (B) DPOAE thresholds were measured in control (blue) and NLRP3^-/-^ (red) mice. The number of mice per group is shown in parentheses. The data represents mean +/- SD.


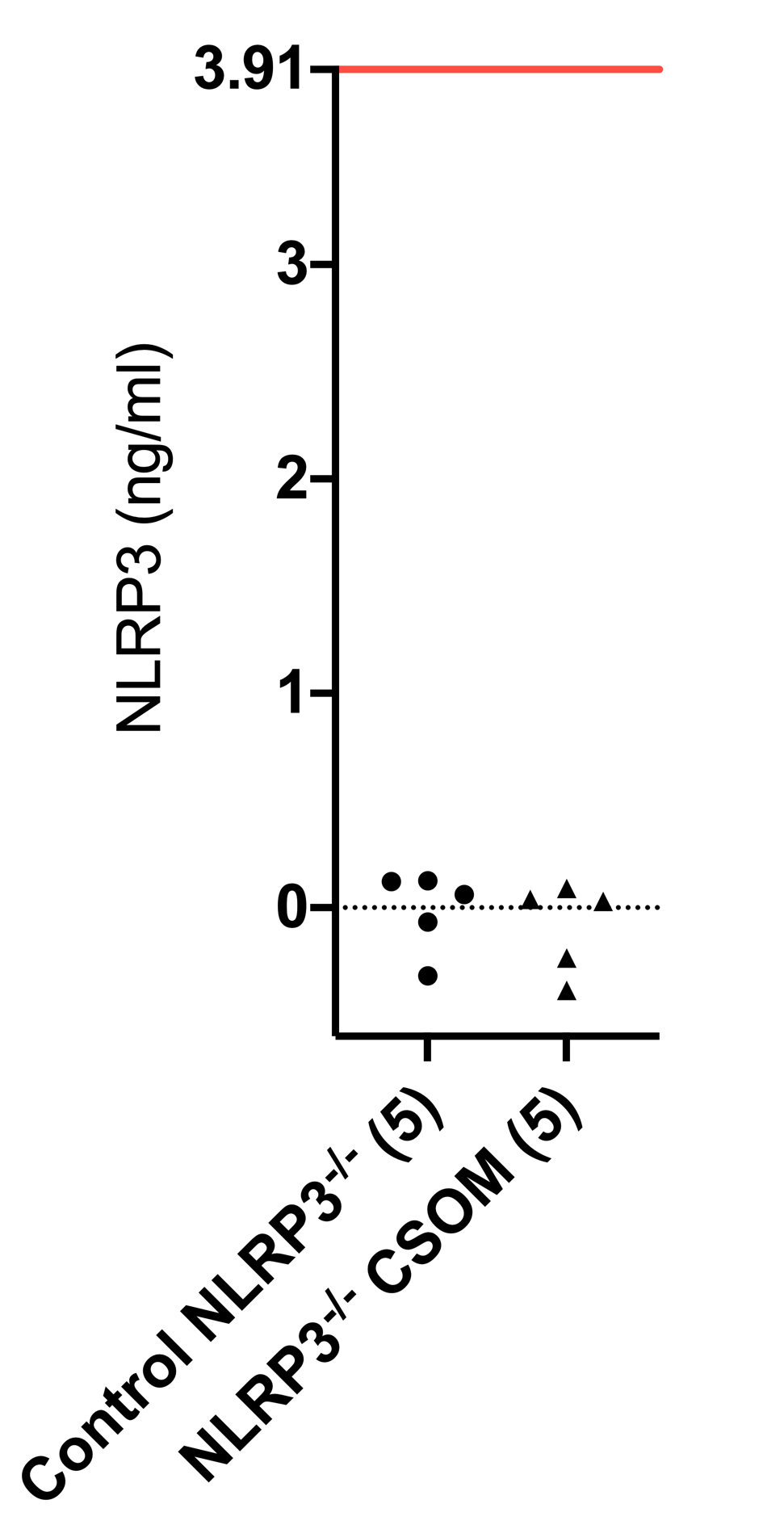


**Fig. S5. NLRP3 is not detectable in cochlear tissues of control NLRP3^-/-^ and NLRP3^-/-^ CSOM mice.** The detectable minimum amount, according to the ELISA kit used, is 3.91 ng/ml, indicated by the red line. The number of mice per group is shown in parentheses.


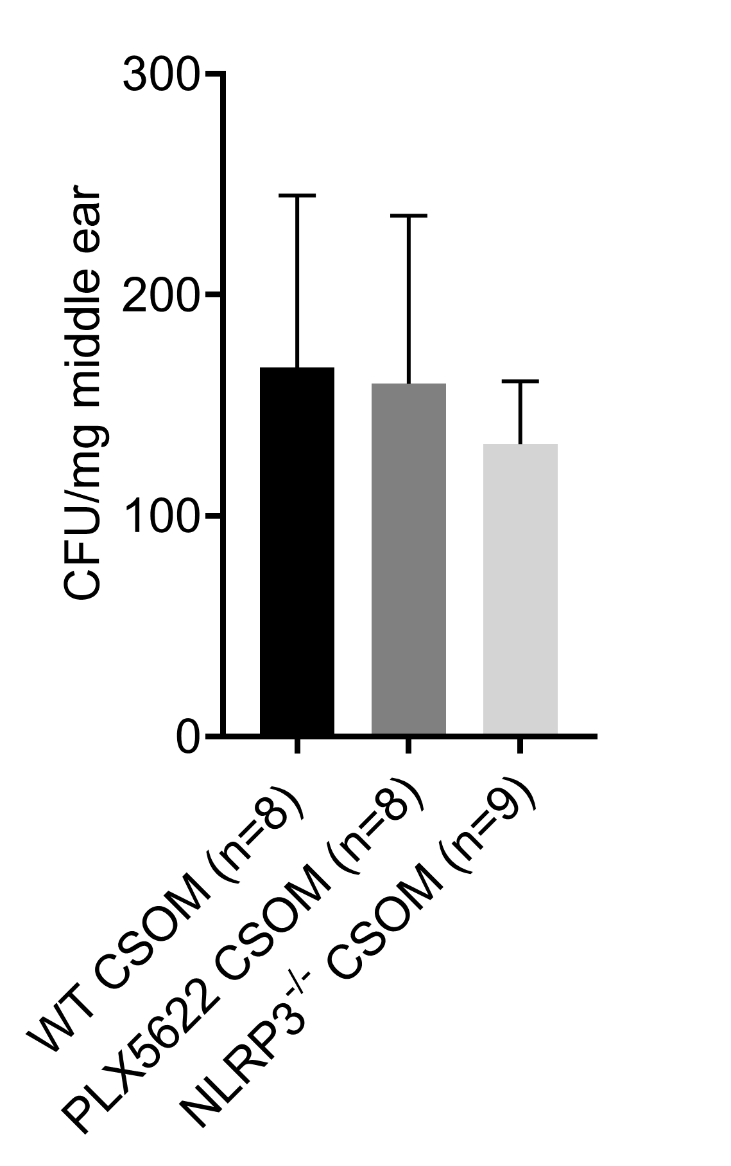


**Fig. S6. CFU count per middle ear weight.**

CFU/mg middle ear was counted at 7d following middle ear infection, with no difference among the groups. The number of mice per group is shown in parentheses. The data represents mean +/- SD.

**
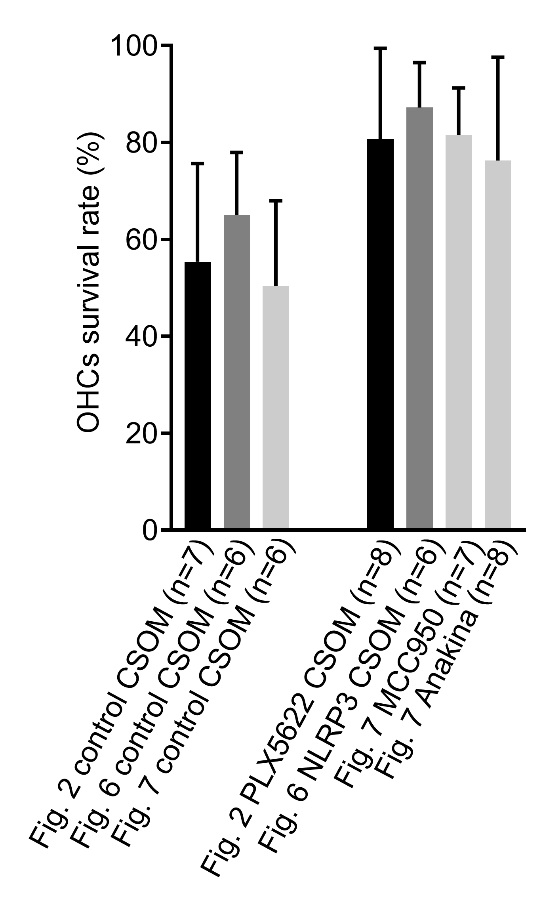
**

**Fig. S7. Comparison of OHC survival rates from Fig. 2, Fig. 6, and Fig. 7.** There are no significant differences among the control CSOM groups (left) or among the treatment and NLRP3 knockout groups in CSOM (right). The number of mice per group is shown in parentheses. Data is shown as mean +/- SD.
